# Strategic Leverage Points in Implementing India's Nikshay Poshan Yojana for Tuberculosis Patients: A Scoping Review

**DOI:** 10.7759/cureus.94143

**Published:** 2025-10-08

**Authors:** Arjun B, Chaitra CM, Ananthakrishnan M, Shemona Gupta

**Affiliations:** 1 Community and Family Medicine, All India Institute of Medical Sciences, Bhopal, Bhopal, IND

**Keywords:** direct benefit transfer (dbt), implementation barriers, implementation research, india, network analysis, nikshay poshan yojana (npy), nutritional support, public health, tuberculosis

## Abstract

Tuberculosis (TB) continues to pose a major public health challenge in India. Malnutrition critically worsens clinical outcomes in patients with tuberculosis. The Nikshay Poshan Yojana (NPY), a national direct benefit transfer (DBT) scheme in India, provides monthly nutritional support to TB patients. The NPY is a flagship program that plays a significant role in India’s fight against TB, and its success depends on effective implementation. This scoping review synthesizes evidence on NPY implementation challenges and possible solutions and models their interconnections using a network approach. Leverage points that can enhance the program's performance are identified through network analysis and quantified via centrality metrics. Following a systematic search of relevant databases, 16 primary research studies evaluating the implementation of NPY were included in the synthesis. Qualitative findings on barriers and solutions were thematically synthesized and modeled as a network, with nodes representing the identified themes and edges indicating the co-occurrence of themes across studies. Degree and betweenness centrality measures identified key leverage points. Four main barrier categories were identified and thematically synthesized: administrative/procedural, financial/banking, patient-level, and healthcare system/provider. These contributed to significant payment delays (median: 43 days to 5.2 months). The network analysis revealed that “lack of bank accounts” was a highly central barrier (degree: 1.0000). Patients' health status also emerged as a critical bridging challenge (betweenness: 0.0604). The key solutions identified included simplifying banking and NPY processes (degree: 0.9286) and implementing a more robust monitoring and evaluation system (betweenness: 0.0482). Interventions targeted at these key leverage points, focusing on improved banking access, streamlined processes, and a strengthened health system, could potentially improve program implementation.

## Introduction and background

Tuberculosis (TB) continues to be a considerable public health crisis globally, with India accounting for over one-fourth of all estimated incident cases worldwide. The bidirectional relationship between malnutrition and TB and the vicious cycle that ensues drive this public health burden: malnutrition increases susceptibility to active TB disease, while TB infection exacerbates undernutrition, leading to adverse treatment outcomes, increased mortality, and higher relapse rates [1,2].

The Government of India launched the Nikshay Poshan Yojana (NPY) in April 2018 as a key component of its National Strategic Plan for Tuberculosis Elimination (2017-2025) [3]. As one of the world’s largest conditional cash transfer initiatives for tuberculosis, this direct benefit transfer (DBT) scheme provides a monthly incentive of ₹1000 (earlier ₹500) to every notified tuberculosis patient for the duration of their anti-tuberculosis treatment (ATT) to improve nutritional status, ensure treatment adherence, and mitigate the catastrophic costs that burden TB care [3-5]. Additionally, the scheme includes provisions for supporting transportation in tribal areas and incentives for treatment supporters and private providers [3].

Wider adoption and effective implementation across diverse settings in India will steer the NPY toward success. While the scheme holds immense potential, early reports and studies have identified various operational challenges that lead to suboptimal implementation and poor outcomes, most critically, significant delays in patients receiving financial support [5]. The NPY operational manual acknowledges the evolving nature of DBT processes and the need for continuous, iterative refinement to streamline operations [3]. A comprehensive understanding of the underlying barriers leading to suboptimal outcomes, program coverage, and fund utilization is essential for policymakers and program managers to refine strategies and optimize implementation. While traditional narrative and thematic reviews can list overarching implementation barriers, they often fail to quantify the complex interplay and relative influence of these factors. Problems such as a patient’s lack of a bank account, technical issues within digital portals (Nikshay and PFMS) [3], or an overburdened healthcare worker are not isolated fragments but interconnected cogs that culminate in delayed service and reduced program effectiveness.

Traditional reviews can list barriers but often fail to reveal the underlying architecture of how these problems sustain each other. This review addresses that gap by applying network analysis, a novel approach that visualizes and quantifies these interconnections [6,7]. By doing so, we move beyond a simple checklist of issues to identify the true systemic leverage points where targeted interventions can create widespread, positive change that will be reflected across multiple stages of program implementation.

## Review

Objectives

This review aims to (i) systematically identify and thematically synthesize the barriers encountered in NPY implementation; (ii) summarize reported NPY outcomes regarding coverage, timeliness of benefit receipt (viewed as a primary outcome reflecting program efficacy), and fund utilization; (iii) identify facilitators and solutions proposed in the literature; and (iv) employ network analysis to map the interconnections between reported barriers and solutions, thereby identifying key leverage points.

Eligibility criteria

This scoping review included primary research studies (qualitative, quantitative, and mixed-methods designs) published in English that focused on NPY implementation in India. To be included, studies had to report on at least one of the following: implementation barriers, facilitators, coverage, or any other outcome measures of the program. Clinical trials focusing on food basket interventions were excluded. Four commentaries discussing NPY implementation were reviewed for contextual insights but excluded from primary data synthesis or network analysis [8-11].

Search strategy

**Figure 1:**
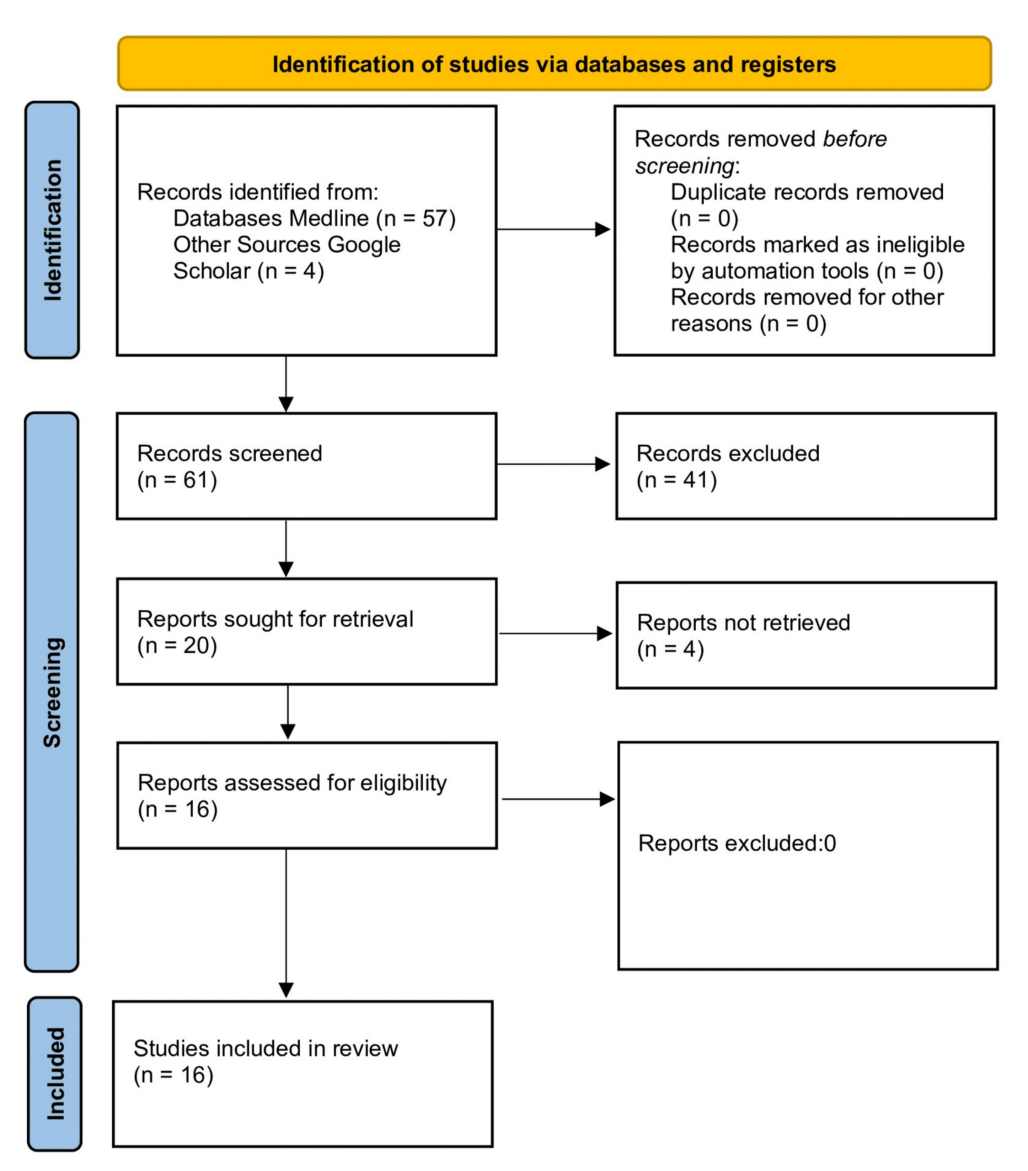
PRISMA-Scr chart PRISMA-Scr: Preferred Report Items for Systematic Reviews and Meta-Analysis Extension for Scope Reviews.

A systematic search was performed in both the MEDLINE database (via PubMed) and Google Scholar for articles published from January 1, 2017, to May 20, 2025. The protocol was prospectively registered in PROSPERO under ID CRD420251069857. The search strategy combined Medical Subject Headings (MeSH) and keywords across three core concepts: (A) the intervention (NPY, DBT, cash transfer, nutritional/financial/social support), (B) the condition (tuberculosis, TB), and (C) the location (India). The reference lists from included articles were hand-searched for additional relevant studies. The initial PubMed search yielded 57 results; after title, abstract, and full-text screening by two independent reviewers, 12 primary articles were included, and an adjudicator resolved any discrepancies. An additional four primary studies were identified through Google Scholar and reference list checking, resulting in a total of 16 primary studies for the review (Figure 1).

Data extraction and quality appraisal

Data from the 16 primary studies were extracted onto a data extraction sheet. Key variables extracted included study design, setting, NPY outcomes, barriers, and solutions as mentioned in the studies. The quality appraisal of the included studies was conducted using the Mixed Methods Appraisal Tool (MMAT) [12]. No studies were excluded; comments from the quality appraisal are included in the Results section.

Synthesis of evidence

Quantitative data on NPY outcomes were descriptively summarized. Qualitative data on barriers and solutions were thematically synthesized after multiple reviews of the studies [3]. Relevant text was collated, and recurring concepts were iteratively mapped to a thematic framework. This process consolidated diverse themes within individual studies into an overarching framework of themes and subthemes.

Network analysis

To visualize and quantify the interconnections between various subthemes, a co-occurrence network was constructed in Python v3.10.16 (Python Software Foundation, Wilmington, Delaware) by mapping keywords and phrases from the studies to the thematic framework. The mapping was refined iteratively to achieve maximum alignment between the extracted data and the framework. In the network, nodes represented distinct thematic barriers or solutions. “Delayed benefit receipt (general system and process delays),” an outcome of these barriers, was included as a central node to analyze its connections. An edge linked two nodes if they were co-reported within the same primary study. The network was condensed to include only nodes reported in two or more studies, resulting in a final network of 29 distinct subthemes (17 barriers, 11 solutions, and the “Delayed benefit receipt” theme) and 310 edges.

NetworkX (v2.8.4, NetworkX Developers, Los Alamos, New Mexico) [13], a Python package, was used to calculate degree centrality and betweenness centrality. Subthemes with high degree centrality are highly connected to other subthemes, while betweenness centrality identifies bridging subthemes that link otherwise distinct subthemes. Barriers with high degree centrality in the network are directly connected to different challenges and may be considered key pressure points that impact multiple facets of the program. Similarly, barriers with high betweenness centrality link otherwise separate clusters of issues; addressing them can prevent failures that may cascade across the system. Nodes ranking high were identified as leverage points. The network was visualized using Plotly (v5.10.0; Plotly, Montreal, Canada).

Results

Description of Included Studies and Quality

Sixteen primary studies (2019-2025) were included in the synthesis [4,5,14-27]. The studies comprised six mixed-methods designs [4,14-16,20,22], five qualitative studies [17-19,25,26], and five quantitative descriptive studies [4,5,21,23,24]. The studies covered diverse settings, ranging from single districts [4,14,20] to multistate evaluations [5,18,19,25]. Participant groups included TB patients, healthcare providers, and program officials. MMAT appraisal indicated that most studies (15/16) had clear research questions, and data collection was appropriate in 14/16. Some qualitative components noted limitations [22], while some quantitative studies reported nonresponse bias [21,27] or limited generalizability [23,26,27].

Key Implementation Barrier Categories

Thematic synthesis identified four overarching barriers consistently hindering NPY implementation (Table 1). First, administrative and procedural hurdles included “Complex, lengthy, or multilevel approval and implementation processes” [18,20,22] and “Nikshay and PFMS portal/software issues (usability, technical, downtime)” [4,5,18,20,25]. Second, financial and banking system bottlenecks included “Lack of bank accounts or difficulty in opening/accessing them (including zero-balance, ID/KYC issues)” as a core issue [4,5,18-20,22,25,26]. Third, patient-level barriers included “Lack of awareness or understanding (NPY scheme, processes, banking)” [5,18,20,23,25-27] and “Perceived inadequacy of benefit amount and associated financial burdens” [18-20,22,25,26]. Finally, healthcare system and provider-level constraints encompassed “Overburdened, inadequate, or lack of dedicated NTEP staff” [5,14,18,20,25] and “Inadequate private sector engagement, support, or interest” [5,24].

**Table 1:** Summary of Main Barrier Categories and Key Predominant Subthemes NPY: Nikshay Poshan Yojana, PFMS: Public Financial Management System, NTEP: National Tuberculosis Elimination Program.

Main Barrier Category	Key Subthemes (Examples From Network Analysis)	Citations
Administrative and procedural hurdles	Complex, lengthy, or multi-level approval and implementation process; Nikshay and PFMS portal/software issues	[4,5,14,16,18-20,22,23,25-27]
Financial and banking system bottlenecks	Lack of bank accounts or difficulty in opening/accessing them; Issues with existing bank accounts; bank-level operational issues	[4,5,14,18-20,22,23,25,26]
Patient-level barriers	Lack of awareness or understanding; perceived inadequacy of benefit amount; patient health status affecting engagement	[5,14-16,18-20,22,23,25,26]
Healthcare system and provider-level constraints	Overburdened, inadequate, or a lack of dedicated NTEP staff; inadequate private sector engagement	[4,5,14,18,20,24,25]

Findings on Coverage, Timeliness, and Utilization

Reported NPY coverage (proportion of notified patients receiving at least one installment) varied significantly. Single-center studies reported rates as low as 28.5% [20], 32.2% [21], 42.2% [14], and 48.4% [26]. Larger or later studies showed higher overall coverage. Analysis of national TB data (2018-2022) showed a coverage of 71.1% [5], 78.5% among HIV-TB coinfected patients in one study [16], 88% in a Gujarat study [15], 88.8% in Chhattisgarh [4], 91% in another Gujarat study [22], and 92.7% (Nikshay data) in a 2022 national survey [25]. One study in North India reported 100% coverage in its limited sample, although it also noted significant delays [27]. A consistent finding was that patients treated in the private sector had significantly lower NPY receipt compared to those in the public sector [5,14,24], as well as lower rates for DR-TB patients [5,24].

Delays in receiving the first NPY installment were a persistent issue across nearly all studies. Median delays from notification or diagnosis to first payment ranged from 43 days [4], 56 days [22], and 74 days [16,20] to 96-105 days [5,25]. One early study reported a median delay of 5.2 months [14]. Studies have reported that a substantial proportion of beneficiaries received their first payment very late in their treatment course [4,16,22] or even after their treatment outcome was declared [16,25]. When the benefit was received, most patients reported utilizing the funds primarily for nutritional support, such as purchasing food, fruits, vegetables, eggs, and groceries [4,17-19,23,25-27]. Some beneficiaries also reported using the funds for other family needs, such as children’s education or daily expenses [4,23]. A consistent theme, however, was the perception among both beneficiaries and some providers that the INR 500 monthly amount was insufficient to meet the nutritional requirements of a TB patient during treatment [18-20,22,25].

Identified Facilitators and Solutions

Across the studies, numerous facilitators and potential solutions were proposed. Systemic and policy enhancements included strong political will [18], simplification of banking norms [4,18], ensuring timely fund flow [14,27], improvements in the digital portals [4,14,18], streamlining approvals [4,18,20,22], and periodically reviewing the benefit amount [18,22]. Improving banking access involved the proactive facilitation of account opening by program staff or NGOs [14,18,22] and leveraging Jan Dhan Yojana accounts [5]. Patient awareness and support could be improved through comprehensive counseling [17,19,27], the design of context-specific IEC materials [19], and accessible grievance redressal mechanisms [18]. Strengthening the health system included periodic updating of staff patterns, capacity building of healthcare providers [14,18,20,22], effective engagement of the private sector [5,14,17,24], improving internet connectivity [4,16,20], and implementing robust real-time monitoring and feedback systems [16,25].

Network Analysis: Systemic Leverage Points

“Delayed benefit receipt (general system and process delays)” was the most central node by degree (1.0000; 12 studies). This was a key outcome measure of the program; it was included in the network to visualize how various barriers and solutions were interconnected with the delay. Key barrier leverage points are detailed in Table 2. “Lack of bank accounts or difficulty in opening/accessing them” (degree: 1.0000; 10 studies) and “Perceived inadequacy of benefit amount and associated financial burdens” (degree: 1.0000; seven studies) were the most connected among barriers. “Patient health status or treatment burden affecting NPY engagement” had the highest betweenness centrality among barriers (0.0604; two studies). Top solution leverage points are shown in Table 3. “Simplify banking norms, policies, and NPY processes” (degree: 0.9286; seven studies) and “Enhance Nikshay/PFMS portals and digital infrastructure” (degree: 0.8571; five studies) were highly connected. “Implement robust M&E and feedback systems for NPY” (betweenness: 0.0482; two studies) showed significant bridging.

**Table 2:** Top Barrier Leverage Points From Network Analysis (n = 29 Nodes) NPY: Nikshay Poshan Yojana, NTEP: National Tuberculosis Elimination Program, PFMS: Public Financial Management System.

Barrier Theme	Main Category	Degree	Betweenness	Article Count	Citations
Delayed benefit receipt (general system and process delays)	Administrative and procedural hurdles	1.0000	0.0000	12	[4,5,14,16,18-20,22,23,25-27]
Lack of bank accounts or difficulty in opening/accessing them (incl. zero-balance, ID/KYC issues)	Financial and banking system bottlenecks	1.0000	0.0118	10	[4,5,14,18-20,22,23,25,26]
Perceived inadequacy of benefit amount and associated financial burdens	Patient-level barriers	1.0000	0.0202	7	[15,18-20,22,25,26]
Lack of awareness or understanding (NPY scheme, processes, banking)	Patient-level barriers	0.9643	0.0144	7	[5,14,16,18,20,23,25]
Overburdened, inadequate, or a lack of dedicated NTEP staff	Healthcare system and provider-level constraints	0.9286	0.0031	6	[4,5,14,18,20,25]
Nikshay and PFMS portal/software issues (usability, technical, downtime)	Administrative and procedural hurdles	0.8929	0.0049	5	[4,5,14,18,20]
Patient health status or treatment burden affecting NPY engagement	Patient-level barriers	0.6071	0.0604	2	[17,19]

**Table 3:** Top Solution Leverage Points From Condensed Network Analysis (n=29 Nodes) NPY: Nikshay Poshan Yojana, PFMS: Public Financial Management System, SNA: Single Nodal Agency, NTP: National Tuberculosis Program, NGO: Non-Governmental Organization, M&E: Monitoring and Evaluation, HR: Human Resources.

Solution Theme	Main Category	Degree	Betweenness	Article Count	Citations
Simplify banking norms, policies, and NPY processes (KYC, Aadhaar, approvals, delivery)	Systemic, Policy, and Portal Enhancements	0.9286	0.0151	7	[4,14,18-20,22,27]
Enhance Nikshay/PFMS portals and digital infrastructure (stability, usability, offline, SNA)	Systemic, Policy, and Portal Enhancements	0.8571	0.0137	5	[4,5,14,20,25]
Facilitate bank account opening and access (NTP/NGOs, Jan Dhan, relatives' accounts)	Improving Banking Access	0.8571	0.0087	4	[5,14,18,22]
Ensure adequate and timely NPY fund flow & review benefit amount	Systemic, Policy, and Portal Enhancements	0.9643	0.0358	6	[14,16,18,19,26,27]
Implement robust M&E and feedback systems for NPY	Strengthening Health System Capacity and Provider Engagement	0.7143	0.0482	2	[16,18]
Strengthen HR (dedicated staff, training, communication, incentives)	Strengthening Health System Capacity and Provider Engagement	0.6429	0.0466	3	[14,18,26]

The network analysis revealed two distinct but equally critical types of implementation barriers. “Lack of bank accounts or difficulty in opening/accessing them” emerged as the primary “super-connector” problem. Its high degree centrality signifies it as the central hub of systemic dysfunction, directly linking to nearly every other barrier in the program. In contrast, “Patient health status or treatment burden” had the highest betweenness centrality, making it the most critical “bridge” problem. This barrier is pivotal not because of its direct connections but because a patient’s poor health connects otherwise separate clusters of issues, such as financial and administrative factors, making it a key point for interventions to prevent failures that may otherwise cascade across the system.

Discussion

This scoping review and the network analysis systematically map the implementation landscape of India's NPY, identifying critical leverage points that could facilitate operational improvements for this conditional cash transfer scheme. Distinct from many global and Indian nutritional support programs that offer in-kind benefits or serve a broader population, the NPY provides direct financial assistance specifically to tuberculosis patients. This targeted approach is crucial for improving nutritional status and supporting long-term treatment adherence. A web of interconnected barriers across administrative, financial, patient, and healthcare system domains hinders the effective implementation of the NPY, potentially compromising its unique benefits. These converge to produce the pervasive outcome of delayed benefit receipt, which was identified as the most central theme in the network, degree centrality (1.0000). The analysis further identified the following barriers as key leverage points, such as the comprehensive "Lack of bank accounts or difficulty in opening/accessing them" (Degree: 1.0000) and "Perceived inadequacy of benefit amount and associated financial burdens" (Degree: 1.0000). Notably, "Patient health status or treatment burden affecting NPY engagement" (Betweenness: 0.0604) emerged as the most critical bridging challenge. Similarly, the network analysis identified solutions such as "Simplify banking norms, policies & NPY processes" (Degree: 0.9286) and system-strengthening solutions like "Implement robust M&E and feedback systems for NPY" (Betweenness: 0.0482) as key leverage points that could be the target of interventions.

The core strength of this review lies in moving beyond thematic listing to exploring interdependencies. Condensing the themes described across multiple studies into a network enhances the robustness of identified core issues. Limitations include reliance on existing published literature, which may not capture the vast spectrum of issues experienced. The keyword-based thematic mapping, though systematic and iteratively refined, might not capture all contextual nuances of human text. Heterogeneity in primary study designs sometimes made the quantitative results unsuitable for pooling.

The findings have direct policy implications. The high degree centrality of "Delayed benefit receipt" demands urgent mitigation of its root causes. First, unblocking banking-related bottlenecks is paramount. The "Lack of bank accounts" barrier had the highest degree centrality (1.0000), identifying it as the most central and interconnected problem in the pipeline of availing the benefit. Therefore, interventions that proactively facilitate bank account opening could have the most significant role in untangling the entire web of challenges [14,18,22,25]. Second, portals and administrative processes require a significant overhaul. The solutions "Enhance Nikshay/PFMS portals & digital infrastructure" and "Simplify banking norms, policies & NPY processes" have a direct impact on highly connected barriers like "Nikshay & PFMS portal/software issues" and "Complex, lengthy, or multi-level approval & implementation process" [4,5,18,20,22,25]. Third, fortifying and capacity building of the health system workforce and its mechanisms is essential. The "Overburdened, inadequate, or lack of dedicated NTEP staff" (Degree: 0.9286) can be addressed by "Strengthen HR" (Betweenness: 0.0466), while "Implement robust M&E and feedback systems for NPY" (Betweenness: 0.0482) can improve accountability [16,25]. Fourth, taking a patient-centered approach by addressing "Lack of awareness" (Degree: 0.9643) and "Perceived inadequacy of benefit amount" (Degree: 1.0000) through better counseling and periodical benefit review is vital [17,20,22,25,27]. The bridging nature of "Patient health status" emphasizes the need for support mechanisms sensitive to patients' ability to navigate the system while ill and to look into a scheme where the benefit amount is graded according to their health status, with a higher benefit for individuals who have severe forms of TB.

Future research should prioritize studies targeting these identified leverage points. Studies utilizing the identified leverage points as an a priori framework could yield deeper insights into causal pathways and the evolving nature of these challenges. The future research should also explore the unique patient experiences and implementation challenges in vulnerable populations, such as those residing in tribal or remote geographical regions.

## Conclusions

India's NPY is a vital nutritional support scheme for TB patients, but its potential is constrained to a great extent by multifaceted implementation barriers that lead to critical delays in benefit delivery. This review, uniquely employing network analysis, has moved beyond thematically listing these issues to the identification of critical systemic leverage points. The findings indicate that interventions focusing on simplifying banking access, enhancing the portals and preventing technical shutdowns, ensuring the adequacy of benefits through timely review, strengthening health system capacity (especially human resources and monitoring and evaluation), and being sensitive to patients' health status offer the most promising pathway to enhancing NPY’s timeliness, coverage, and ultimate public health impact in India’s fight against tuberculosis.
